# Evaluation of Feed Near-Infrared Reflectance Spectra as Predictors of Methane Emissions from Ruminants

**DOI:** 10.3390/ani12182478

**Published:** 2022-09-19

**Authors:** Xuezhao Sun, David Pacheco, Grant Taylor, Peter H. Janssen, Natasha M. Swainson

**Affiliations:** 1 AgResearch Limited, Grasslands Research Centre, Palmerston North 4442, New Zealand; 2 The Innovation Centre of Ruminant Precision Nutrition and Smart and Ecological Farming, Jilin Agricultural Science and Technology University, Jilin City 132109, China; 3 Jilin Inter-Regional Cooperation Centre for the Scientific and Technological Innovation of Ruminant Precision Nutrition and Smart and Ecological Farming, Jilin City 132109, China

**Keywords:** feed, methane, NIRS, prediction, ruminant

## Abstract

**Simple Summary:**

Methane is a greenhouse gas and its emissions contribute to global warming. Domestic farmed ruminants are one of the major contributors to anthropogenic methane emissions. Feed consumed by ruminants produces methane when fermented by the rumen microbiota. Thus, feed chemical composition could influence the amount of methane produced per unit of feed eaten (i.e., methane yield). Near-infrared reflectance spectroscopy (NIRS) is commonly used to estimate feed chemical composition by correlating dietary constituents against features of the near-infrared reflectance (NIR) spectrum of the feed. Thus, NIRS might be able to predict methane yield. Feed samples collected from sheep and cattle experiments in which methane was measured were scanned for NIR spectra. These spectra and methane data were used to establish prediction models. The modeling results suggested that 53% of the variation in methane yield can be predicted using NIRS. The accuracy of the prediction is modest, but it could be still useful for screening low methane feeds. To increase the accuracy of the prediction, we recommend that more data from animal experiments with measurements of methane emissions are included in the databases for NIRS calibrations and alternative algorithm methods and combination of other techniques to NIRS should be explored.

**Abstract:**

Feed chemical composition is associated with methane (CH_4_) formation in the rumen, and thus CH_4_ yields (Y*_m_*; CH_4_ emitted from per unit of dry matter intake) could be predicted using near-infrared reflectance spectroscopy (NIRS) of feeds fed to ruminants. Two databases of NIRS data were compiled from feeds used in experiments in which CH_4_ yields had been quantified in respiration chambers. Each record in the databases represented a batch of feed offered to a group of experimental animals and the mean CH_4_ yield for the group. A near-infrared reflectance spectrum was obtained from each feed, and these spectra were used to generate a predictive equation for Y*_m_*. The predictive model generated from brassica crops and pasture fed at a similar feeding level (*n* = 40 records) explained 53% of the variation in Y*_m_* and had a reasonably good agreement (concordance correlation coefficient of 0.77). The predictive ability of the NIRS calibration could be useful for screening purposes, particularly for predicting the potential Y*_m_* of multiple feeds or feed samples, rather than measuring Y*_m_* in animal experiments at high expenses. It is recommended that the databases for NIRS calibrations are expanded by collecting feed information from future experiments in which methane emissions are measured, using alternative algorithms and combining other techniques, such as terahertz time-domain spectroscopy.

## 1. Introduction

Methane (CH_4_) is one of the major greenhouse gases (GHG) contributing to global warming [1]. From 1900 to 2021, the CH_4_ concentration in the atmosphere has more than doubled, from 862 to 1842 parts per billion (ppb) [2] and its potential to contribute to global warming is 28 times greater than carbon dioxide [3]. Globally, about 500–600 million tonnes of CH_4_ are emitted into the atmosphere each year [4]. Much (40%) of these emissions comes from livestock industries [5], accounting for approximately 6% of total anthropogenic GHG emissions [6,7]. Demands from society for the mitigation of CH_4_ emissions resulted in the Global Methane Pledge in November 2021 in Glasgow and signed by over 110 countries (https://www.globalmethanepledge.org/, assessed on 30 July 2022). In addition to the global warming effect, 3.9–10.7% of ingested metabolic energy could be lost as CH_4_ emissions from ruminants [8]. The mitigation of CH_4_ emissions from ruminants thus not only benefits the environment, but also has the potential to improve animal productivity.

Methane is formed as a by-product from the degradation and fermentation of the feed in the rumen, with a small amount of CH_4_ also produced in the hindgut [9,10]. There is a consensus that feed quality is a key attribute of the methanogenic potential of a feed, particularly for feeds such as conserved forages and concentrates [11]. In general, it is accepted that diets rich in structural carbohydrates (i.e., fibrous, low-quality feeds) lead to greater methane yields [Y*_m_*: yield of CH_4_ per unit of dry matter intake (DMI)] than feeds with low structural carbohydrates, as a result of a slower rate of fermentation and longer retention time of the feeds in the rumen [12]. However, for fresh forage diets, the influence of chemical composition on Y*_m_* is equivocal. On the one hand, a meta-analysis of experiments conducted in New Zealand with ryegrass-based pastures suggested that chemical composition variables only account for a small proportion (20%) of the variation in Y*_m_* [13]. On the other hand, dietary attributes, such as organic matter digestibility (OMD) and neutral detergent fiber, have been postulated as good predictors (R^2^ = 0.77) of Y*_m_* from legumes and forages [14]. Dietary attributes present in fresh forages, such as nitrates [15], lipids [16,17,18] and tannins [15,19], have been associated with reductions in Y*_m_*. An evaluation of experiments conducted in respiration chambers has described negative correlations between OMD and methane emissions per unit of digestible organic matter intake across a variety of fresh forages fed to sheep [20], which explain 48% and 64% of the variation in Y*_m_* from individual and group mean data, respectively.

Near-infrared reflectance spectroscopy (NIRS) is a method for estimating the chemical composition of feeds. NIRS predictions rely on correlating a known dietary constituent, normally measured using wet chemistry methods, against features of the near-infrared reflectance (NIR) spectrum of the feed. NIRS has been used to estimate the ‘conventional’ nutrient composition (e.g., crude protein, neutral detergent fiber, etc.) [21] of feed, but it has also proven useful to predict concentrations of compounds such as tannins and non-starch polysaccharides [22,23], or to predict properties of a feed, such as digestibility or rumen degradability, which are observed after a feed is offered to animals [23,24,25]. NIRS has also shown a potential to estimate the yield of CH_4_ from biomass digestors for biogas production [26,27] and has been successful in predictions of CH_4_ production during rumen batch culture fermentation with over 100 forage species [28].

An NIRS calibration developed for Y*_m_* could provide an alternative for high throughput identification of low-CH_4_ feeds, to complement animal experiments and statistical and mechanistic predictive models. It could also be used to assess Y*_m_* from different feeding systems, if these were known to vary and shown to be predicted by NIRS, without having to test each in animal experiments.

The objective of this study was to conduct an initial assessment of the potential of NIRS to predict Y*_m_* from ruminants consuming different feeds. The concept is based on the proven ability of NIRS to identify feed characteristics. One of the biggest advantages of NIRS is that many composition parameters, including complex features, can be predicted with a single NIR spectrum acquisition [26]. Therefore, we hypothesized that a naïve analysis (i.e., without any prior knowledge of feed characteristics affecting Y*_m_*) could capture simultaneously all feed features that influence CH_4_ formation in the rumen of livestock. From this initial assessment, we expected to determine the usefulness of conducting further analyses, by including additional samples (archived or fresh) to expand calibration datasets. Typically, a working NIRS calibration requires several hundred independent samples representing a variety of seasons, years and feed types, to establish a useable calibration curve.

## 2. Materials and Methods

### 2.1. Data

The databases were formed from a mix of newly scanned samples with associated CH_4_ emissions data and historical spectra for which a positive identification to an animal experiment with associated CH_4_ emissions data could be made. Samples that had been stored for > 2 years were not analyzed by NIRS due to potential sample deterioration. This criterion was based on comparisons of historical and new spectra from samples stored for different lengths of time. Methane emissions were measured over 48 h in open-circuit respiration chambers for sheep and cattle at a single location [29].

Two databases were assembled to evaluate the potential of NIRS as a high throughput predictor for Y*_m_*. The first database (DB1) consisted of records associated with 40 feed samples (30 brassica crops, 10 ryegrass pastures). Each feed containing one forage or ryegrass pasture alone or a mixture of one brassica crop and ryegrass pasture had been fed to a group of experimental sheep or cattle (*n* = 2–14 per group) and the feed was collected and pooled over the CH_4_ measurement period for NIR spectra. The CH_4_ measurements were conducted at the New Zealand Ruminant Methane Measurement Centre in Palmerston North New Zealand [30,31,32,33] (Sun and Pacheco, unpublished) and the mean Y*_m_* of a group of experimental sheep or cattle was entered as an observation in the database. The level of feeding was similar [1.6 × metabolic energy (ME) maintenance requirements] for all the animals. It is generally accepted that feeding level can influence Y*_m_* [34,35]. Therefore, a database of experiments at a similar feeding level was deemed appropriate to determine the value of NIRS prediction without the confounding effect of feeding level, which is obviously not associated with spectral features. A summary of the records included in this database is presented in Table 1.

The second database (DB2) consisted of DB1 plus 25 records from four experiments with sheep in respirations chambers with NIRS analysis previously performed. The additional records included data from a wider variety of grass types, plus white clover. A description of DB2 is given in Table 2. This database included experiments in which a wider range of feeding levels were used (mean 1.8, range 1.6 to 1.9 multiples of ME maintenance requirements).

In the expanded database (DB2), there was a dominance of ryegrass samples and forage rape, with a few records for a variety of other pasture species. The database expanded the range of Y*_m_* for grasses and included records from forage samples across the period 2009–2014.

### 2.2. Generation of NIR Spectra and Calibration

Thirty-eight feed samples were scanned as part of this study and included in DB1. The samples were oven dried at 65 °C and finely ground to pass a 1-mm sieve before being scanned over the wavelength range of 400 to 2500 nm to obtain NIR spectra. The rest of the feed samples (2 in DB1 and 25 in DB2) had archived spectra from previous scans over the same wavelength range. All samples were scanned using a Bruker MPA Fourier-transform NIR spectrophotometer (BrukerOptik GmbH; Ettlingen, Germany) and the resulting NIR spectra were analyzed using Optic User Software (OPUS) version 7.0 (BrukerOptik GmbH; Ettlingen, Germany).

From the spectra, naïve calibrations were generated between the NIR spectra and CH_4_ yield (g CH_4_/kg DMI). The calibration models were based on principal component analysis and cross-validation using the most appropriate mathematical treatment for the spectra of each sample set; either first derivative, vector normalization or in some cases a combination of both [36]. Predicted values were obtained from each feed spectrum using the OPUS software.

### 2.3. Evaluation of the Prediction of Y_m_

Predicted values from the NIRS analyses were compared to the observed (experimental treatment mean) values using regression analysis, concordance correlation coefficient analysis [37] and error decomposition analysis [38]. Additional evaluation used the residual prediction deviation (RPD), which is the ratio of the standard deviation of the observed values to the squared root of the mean prediction error (RMSPE). RPD is a parameter to assess the goodness of the prediction relative to the inherent variability of the variable to be predicted [39]. Larger values of RPD indicate that the error of the model is smaller relative to the variation in observed Y*_m_*.

## 3. Results and Discussion

Two sets of results are presented. The first set of results is from analyses of data in DB1, which had records at a common feeding level. The second set of results is from analysis of data in the larger dataset DB2, which included experiments over a range of feeds and feeding levels.

### 3.1. Results from DB1 with Common Feeding Level

**Figure 1:**
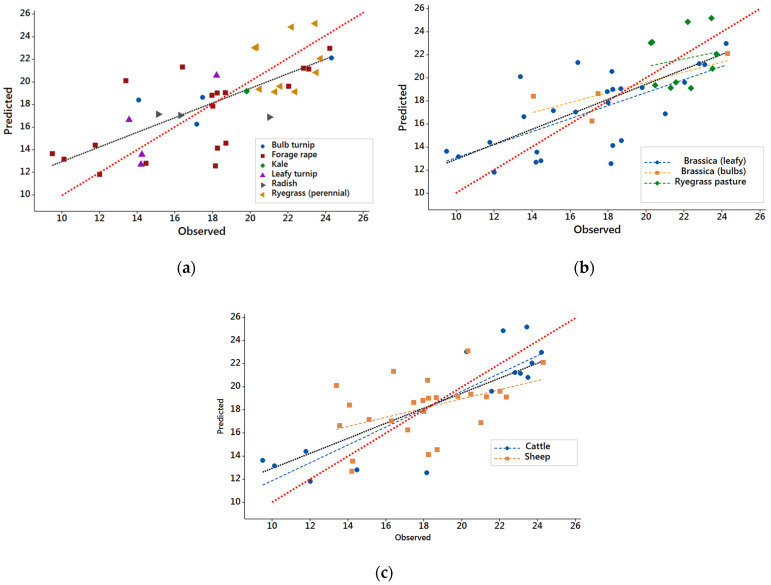
Scatterplots of the observed methane yields and methane yields predicted from near-infrared reflectance spectra for samples in database DB1 [all experiments with a common feeding level of 1.6× metabolizable energy (ME) maintenance requirements]. The red dotted line in all plots is the line predicted = observed. The black dotted line in all plots represents the regression predicted = 6.45 + 0.649 (observed); adjusted R^2^ = 0.53; both the intercept and slope are significantly different from zero: *p* < 0.001. (**a**) Scatterplot of all samples in database DB1. (**b**) Scatterplot according to feed type, for samples in database DB1. The blue, orange and green dotted lines represent the regression lines for leafy brassicas, brassica bulbs and ryegrass pasture, respectively. Only leafy brassicas had regression parameters significantly different from zero: *p* < 0.001. (**c**) Scatterplot according to animal species, for samples in database DB1. The blue line and dark red dotted lines represent the regression lines for cattle and sheep, respectively. Both regression lines are significant *p* < 0.01.

A calibration was generated from DB1. Using this calibration, predictions were generated and compared against the observed Y*_m_* values. Overall, the predictions of Y*_m_* from the best calibration obtained from the spectra of samples in DB1 had a significant, positive correlation with the observed values of Y*_m_* (Figure 1a).

Further graphical analysis was conducted to determine if the overall regression between predicted and observed values in DB1 was influenced by feed type (Figure 1b) or animal species (Figure 1c).

When the data were analyzed by forage type, only the regression parameters for leafy brassicas were significantly different from zero. The non-significant regressions can be attributed to the small range of Y*_m_* for ryegrass pasture, and the small number of observations for brassica bulbs. Therefore, the analysis of the prediction for feed type was limited to leafy brassica crops.

When the database was separated by animal species, the regression parameters describing the association between the observed Y*_m_* and predicted Y*_m_* from NIRS were significant for both sheep and cattle. Further analysis determined that the slopes of the regression lines for each species were not different from each other (95% confidence intervals: 0.536–1.007 and 0.063–0.737 for cattle and sheep, respectively). The lack of evidence for a difference in slopes between animal species suggests the potential for having a single calibration for both cattle and sheep. However, it would be desirable to include data for cattle with intermediate values of Y*_m_*, because the current dataset consists of two discrete data groups for cattle at both ends of the Y*_m_* range, in which the low values were from cattle fed forage rape and the high values from cattle fed ryegrass pasture. Sheep data were more uniformly represented across the range of Y*_m_* and included a larger variety of feeds. Because of the distribution of observed values for cattle, no further analysis of the cross-validation at the animal species level was attempted.

### 3.2. Results from DB2

**Figure 2:**
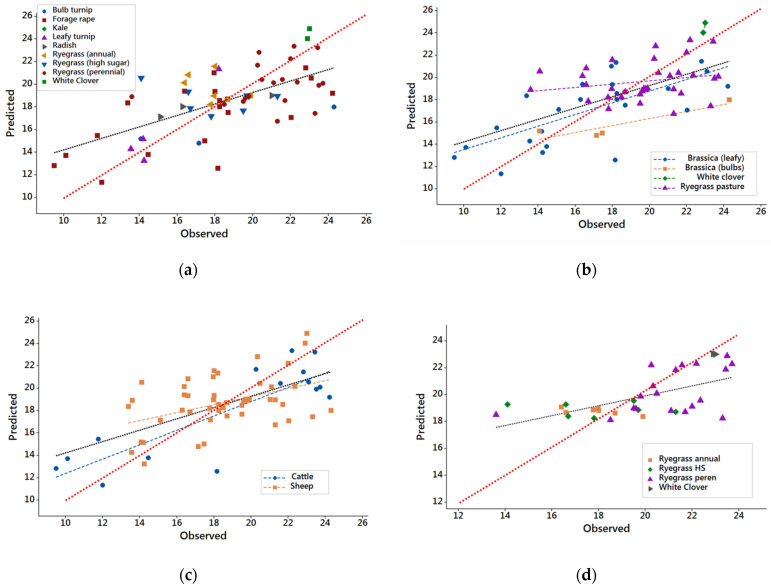

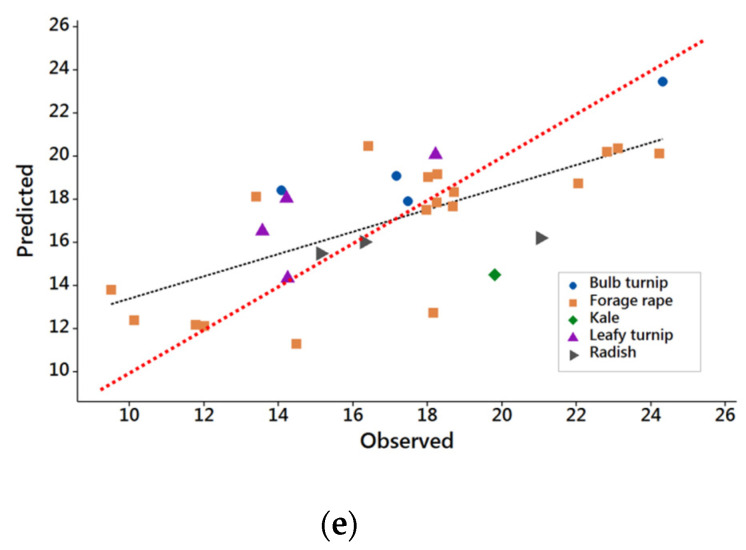
Scatterplots of the observed methane yields and methane yields predicted from near-infrared reflectance spectra for samples in expanded database DB2. The red dotted line in all plots is the line predicted = observed. The black dotted line in plots (**a**–**c**) represents the regression predicted = 9.17 + 0.506 (observed); adjusted R^2^ = 0.40; both the intercept and slope are significantly different from zero (*p* < 0.001). (**a**) Scatterplot from all samples. (**b**) Scatterplot according to feed type, for samples in expanded database DB2. The blue, orange, green and purple dotted lines represent the regression lines for leafy brassicas, brassica bulbs, white clover and ryegrass pasture, respectively. Only the regression line for leafy brassicas is significantly different from zero (*p* < 0.001). (**c**) Scatterplot according to animal species. The blue and orange dotted lines represent the regression lines for cattle and sheep, respectively. Both regression lines are significantly different from zero (*p* < 0.01). (**d**) Scatterplot from ryegrass samples. The black dotted line represents the regression predicted = 12.53 + 0.369 (observed); adjusted R^2^ = 0.34; both the intercept and slope are significantly different from zero (*p* < 0.001). (**e**) Scatterplot from brassica samples. The black dotted line represents the regression predicted = 8.21 + 0.518 (observed); adjusted R^2^ = 0.44; both the intercept and slope are significantly different from zero (*p* < 0.001).

The predictions from the model generated from records in the extended DB2 were significantly and positively correlated to the observed values (adjusted R^2^ = 0.40). The inclusion of the additional records resulted in an expanded range in Y*_m_* for ryegrass pastures, through including different species (annual ryegrass) and cultivars (‘high-sugar’ grass) (Figure 2a).

Even with the larger number of records in DB2, only the parameters for the regression line for brassicas were significantly different from zero (Figure 2b). However, the regression line for white clover could be estimated because only two records were available for this feed.

The regression line for leafy brassicas using records in DB2 [predicted = 8.26 + 0.526 (observed); adjusted R^2^ = 0.48] (Figure 2b) was similar to the regression line describing the relationship for the whole dataset (DB2) [predicted = 9.17 + 0.506 (observed)]. Even with the larger range in Y*_m_* for ryegrasses in DB2, the regression between predicted and observed values was not significantly different from zero. As expected, the regression between observed Y*_m_* and predicted Y*_m_* for bulb brassicas was not significant, as described previously for DB1 (all bulb brassica records were included in the DB1, since no additional records for this forage type were in DB2).

When the scatterplot of predicted versus observed values was analyzed using animal species as a group (Figure 2c), the analysis of regression lines supported the results obtained from DB1, namely that the regression slopes were not different between animal species (95% CI: 0.436 − 0.847 and 0.140 − 0.577 for cattle and sheep, respectively). However, DB2 did not contain additional records for cattle (Table 2), so no additional observations covering the mid-range of Y*_m_* for cattle data were able to be added.

Additional models were generated for subsets of DB2, to generate feed specific predictions. These models were generated for brassica crops (35 records) and grass pastures (30 samples). The pasture-specific model resulted in a regression line with parameters significantly different from zero [predicted = 12.5 + 0.369 (observed); *p* < 0.001], but with moderate explanatory power (R^2^ = 0.34; Figure 2d). It is important to note the presence of observations (one perennial ryegrass and one high-sugar grass) that strongly influence the regression line. The brassica-specific model also had a significant regression line [predicted = 8.21 + 0.518 (observed); *p* < 0.001], with slightly higher explanatory power (R^2^ = 0.44; Figure 2e).

### 3.3. Evaluation of NIRS Calibrations

Further analysis of the four models developed (Table 3) indicated that the NIRS predictive model for Y*_m_* had a very small mean bias, and small slope bias with random error contributing to most (>95%) of the residual prediction error. The relative prediction error [RPE: root mean squared prediction error (RMSPE) expressed as a proportion of the mean] was below 17%, which could be considered as moderately adequate [40]. Furthermore, the concordance correlation coefficient (CCC; a measure of the agreement between two variables: i.e., line *x* = *y*) was acceptable (0.73) for the model developed for all feeds from DB1, but only moderate (CCC 0.53 to 0.65) for the other three models. Of the four models, the one developed for all feeds from DB1 had the greatest coefficient of determination (R^2^ = 0.53), which measures the proportion of the variance explained by the model, while the remaining models explained between 34 to 44% of the variance (R^2^ = 0.34 and 0.44, respectively). Finally, the models were evaluated in terms of their residual prediction deviations (RPD), a common statistic to assess NIRS calibrations. The model for all feeds generated from DB1 had the greatest RPD value of the models developed (1.47), which indicates a calibration that could be considered useful for screening purposes, particularly when taking into account the small number of records (for a NIRS calibration). Ideally, the models should have R^2^ values greater than 0.7 and RPD values greater than 1.75 to be considered ‘moderately useful’ for prediction purposes [41].

For comparison purposes, the best multiple regression model (four predictor variables) in the analysis reported by Hammond et al. [13] could only explain half as much (R^2^ = 0.20) of the variance of Y*_m_* in their considerably larger database (*n* = 161 records). This difference illustrates the potential of NIRS as a tool to explore yet-to-be-identified components (or interactions between components) in the feed, which could help explain differences between Y*_m_* from feeds.

Although the statistics for this predictive model seem modest, it is important to note some of the factors contributing aspects to this. Most NIRS calibrations require > 100 samples to be defined to be useful. For CH_4_ generation potential from biofuels feedstocks, NIRS has provided satisfactory calibrations with R^2^ values > 0.80 and RPD values > 2.4 [26,27], but with larger datasets (296 and 88 samples per study, respectively). These studies provide evidence that a feature such as CH_4_ yield, resulting from the interaction of feed and microbial fermentation, can be adequately predicted from feed NIRS analysis. However, in contrast to biogas digesters, which have relatively constant input rates and stable conditions, CH_4_ formation in the rumen results from more heterogeneous inputs and conditions. This difference may also partly explain the modest predictive ability of the models presented here. Another aspect, which is supported by the less accurate predictions obtained from the larger database (in spite of the larger number of samples), is the fact that Y*_m_* is affected by feeding level. NIRS will be unable to make any distinction for the spectra for animal experiments at different feeding levels, as this information is not intrinsic to the NIR spectra. However, it could provide data that are comparable at a given feed intake, given an assessment of CH_4_ emissions potential from a feed rather than generating a prediction for a given animal on a feed.

The analysis of models generated for sheep and cattle suggests that a single curve to predict Y*_m_* for both species may be feasible. However, these initial calibrations were able to explain a larger proportion of variation in Y*_m_* than previous statistical attempts for comparable diets [13]. Overall, the analyses made here suggest that further development of NIRS for predicting Y*_m_* could generate a valuable tool. Expansion of this database could be achieved by including routine NIRS scanning from animal experiments in which methane emissions are measured, including the use of suitable archived samples. The expense for doing this is small and would not involve a large deviation to typical protocols for existing animal experiments. This expansion of the databases would enable testing for improvement of the correlation and RPD values. Although NIRS has been used extensively to predict methane production in biogas production [42] and in rumen batch culture fermentation [28], to the best of our knowledge this is the first time to have calibrations between feed NIR spectra and CH_4_ yields measured from animal experiments. The calibrations could be improved by using alternative algorithms [43] and combining other techniques, such as terahertz time-domain spectroscopy [44], in addition to the expansion of the database.

## 4. Conclusions

Based on the results presented, it is feasible that a usable calibration curve could be generated between NIR spectra and CH_4_ yield, particularly with inclusion of a larger number and variety of samples.

## Figures and Tables

**Table 1:** Description of the database DB1, showing forage type and animal species.

Forage Type	Animal Species		Mean Y*_m_*	Range Y*_m_*	Total (*n*)
	Cattle (*n*)	Sheep (*n*)			
Bulb turnip (*Brassica campestris*)		4	18.2	14.1–24.3	4
Perennial ryegrass (*Lolium perenne*)	6	4	21.9	20.3–23.7	10
Kale (*B. oleracea*)		1	19.8	19.8	1
Leafy turnip (*B. campestris*)		4	15.1	13.6–18.2	4
Forage radish (*Raphanus sativus*)		3	17.5	15.1–21.0	3
Forage rape (*B. napus*)	9	9	17.1	9.5–24.2	18
Mean Y*_m_*	18.7	18.1			
Range Y*_m_*	9.5–24.2	13.4–24.3			
Total	15	25	18.3	9.5–24.3	40

The database contained records for methane yield (Y*_m_*; g CH4/kg dry matter intake) from experiments in which animals were fed at 1.6 × metabolic energy maintenance requirements.

**Table 2:** Description of the expanded database DB2, showing forage type and animal species.

Forage Type	Animal Species		Mean Y*_m_*	Range Y*_m_*	Total (*n*)
	Cattle (*n*)	Sheep (*n*)			
Bulb turnip (*Brassica campestris*)		4	18.2	14.1–24.3	4
Kale (*B. oleracea*)		1	19.8	19.8	1
Leafy turnip (*B. campestris*)		4	15.1	13.6–18.2	4
Forage radish (*Raphanus sativus*)		3	17.5	15.1–21.0	3
Forage rape (*B. napus*)	9	9	17.1	9.5–24.2	18
High sugar ryegrass (*Lolium perenne*)		7	17.9	14.1–21.3	7
Perennial ryegrass (*L. perenne*)	6	13	21.0	13.6–23.7	19
Annual ryegrass (*L. rigidum*)		7	17.0	16.4–19.9	7
White clover (*Trifolium repens*)		2	23.0	22.9–23.0	2
Mean Y*_m_*	18.7	18.6			
Range Y*_m_*	9.5–24.2	13.4–24.3			
Total	15	50	18.6	9.5–24.3	65

The database contained all records for methane yield (Y*_m_*; g CH_4_/kg dry matter intake) from experiments in which animals were fed an extended range of forage and at extended feeding levels.

**Table 3:** Evaluation of the predictive models of methane (CH_4_) yield (Y*_m_*) generated from near-infrared reflectance spectra of feed samples from experiments in which animals were fed at 1.6 × metabolizable energy (ME) maintenance requirements. Statistics compare the performance of the model to predict Y*_m_* across all feed types in the database.

	Y*_m_* (g CH_4_/kg DMI ^a^)		Y*_m_* (g CH_4_/kg DMI)	
	Database DB1	Database DB2	Brassica Only Model	Pasture Only Model
Data processing	VN ^b^	FD ^c^/VN	FD/VN	FD
Number of samples (*n*)	40	65	30	35
Mean observed	18.31	18.59	17.10	19.86
Mean predicted	18.35	18.57	17.07	19.85
Mean bias	0.04	−0.02	−0.03	−0.01
RMSPE ^d^	2.77	2.77	2.88	2.08
Relative prediction error ^e^	15.1	14.9	16.9	10.5
Error decomposition:				
% bias	0.02	0.01	0.01	0.01
% slope	4.4	3.9	1.4	0.05
% random	95.6	96.1	98.6	99.95
Adjusted R^2 f^	0.53	0.40	0.44	0.34
CCC ^g^	0.73	0.62	0.65	0.53
RPD ^h^	1.45	1.28	1.35	1.25

^a^ DMI = dry matter intake; ^b^ VN = vector normalization; ^c^ FD = first derivative; ^d^ RMSPE = Root mean squared prediction error; ^e^ Root mean square prediction error/Mean observed × 100; ^f^ R^2^ of the regression predicted versus observed; ^g^ Lin’s concordance correlation coefficient; ^h^ Residual prediction deviation (SD/RMSPE).

## Data Availability

On reasonable request, the original databases for modeling are available after approval by this project’s funders.
